# Facile Synthesis of Porous Ag Crystals as SERS Sensor for Detection of Five Methamphetamine Analogs

**DOI:** 10.3390/molecules27123939

**Published:** 2022-06-20

**Authors:** Yazhou Qin, Fan Mo, Sen Yao, Yuanzhao Wu, Yingsheng He, Weixuan Yao

**Affiliations:** 1 Key Laboratory of Drug Prevention and Control Technology of Zhejiang Province, Zhejiang Police College, 555 Binwen Road, Binjiang District, Hangzhou 310053, China; yazhouqin@zju.edu.cn (Y.Q.); mofan@zjpc.edu.cn (F.M.); yaosen@zjpc.edu.cn (S.Y.); wuyuanzhao@zjpc.edu.cn (Y.W.); 2 Key Laboratory of Drug Control and Monitoring, National Anti-Drug Laboratory Zhejiang Regional Center, 555 Binwen Road, Binjiang District, Hangzhou 310053, China

**Keywords:** Ag crystal, methamphetamine, SERS, porous structure

## Abstract

Porous noble metal nanomaterials have attracted extensive attention due to their high specific surface area and surface plasmon resonance effect. However, it is difficult to form porous structures due to the high mobility and low reduction potential of noble metal precursors. In this article, we developed a facile method for preparing porous Ag with a controllable structure at room temperature. Two kinds of Ag crystals with different porous structures were successfully prepared by using AgCl cubes as sacrificial templates. Through the galvanic replacement reaction of Zn and AgCl, Ag crystals with a sponge-like porous structure were successfully prepared. Additionally, using NaBH_4_ as the reducing agent, we prepared granular porous Ag cubes by optimizing the amount of reducing agent. Both the sponge-like and granular porous Ag cubes have clean and accessible surfaces. In addition, we used the prepared two porous Ag cubes as substrate materials for SERS detection of five kinds of methamphetamine analogs. The experimental results show that the enhancement effect of granular porous Ag is better than that of sponge-like porous Ag. Furthermore, we probed the hot spot distribution of granular porous Ag by Raman mapping. By using granular porous Ag as the substrate material, we have achieved trace detection of 5 kinds of methamphetamine analogs including Ephedrine, Amphetamine, N-Methyl-1-(benzofuran-5-yl)propan-2-amine (5-MAPB), N-Methyl-1-(4-methoxyphenyl)propan-2-amine (PMMA) and N-Methyl-1-(4-fluorophenyl)propan-2-amine (4-FMA). Furthermore, to achieve qualitative differentiation of analogs with similar structures we performed density functional theoretical (DFT) calculations on the Raman spectra of the above analogs. The DFT calculations provided the vibrational frequencies, Raman activities, and normal mode assignment for each analog, enabling the qualitative differentiation of the above analogs.

## 1. Introduction

Noble metal nanomaterials have aroused great interest among researchers due to their excellent localized surface plasmon resonance (LSPR) characteristics. Its unique LSPR characteristics are widely used in catalysis, surface-enhanced Raman scattering (SERS), photothermal therapy, and chemical and biological sensing technologies [1,2,3,4,5]. Due to the fact that the LSPR effect of noble metal nanoparticles is closely related to their morphology and size, a lot of research has been devoted to regulating the morphology and size of noble metal nanoparticles in the past few decades. Many noble metal nanoparticles with regular morphology and uniform size, including spherical [6,7], octahedral [8,9], cubic [10,11], rhombic [12,13], rod-shaped [14,15], plate-shaped [16,17], and high-index facet nanoparticles [18,19,20], have been prepared. In addition, in the application field of SERS, noble metal nanoparticles with sharp tips or porous structure usually show more excellent effects. In recent years, numerous studies have reported on the preparation methods of noble metal nanoparticles with sharp tips or nano-gaps, including nanostars [21,22], nanoflowers [23,24], and porous nanoparticles [25,26].

In particular, porous structures have attracted intensive attention due to their large relative surface area, high internal “hot spot” density, and optical properties related to porosity. Due to their unique structure-related properties, porous structures have been widely used in various research fields, including catalysis [27,28,29], sensing [30,31], and SERS [32,33,34,35]. As a kind of porous material, porous noble metal material not only has a high surface area, high gas permeability and low density, but also has surface plasmon resonance characteristics, which has a wide range of applications in the optical field [36,37,38]. For example, Liu et al. deposited a layer of gold on a hexagonal densely stacked polystyrene (PS) colloidal monolayer by plasma sputtering, and then obtained an ordered array of Au open-nano-shells after removing the PS colloidal template. By changing the experimental parameters, the SPR characteristic could be regulated to the near infrared region and realize near infrared SERS highly sensitive detection [36]. Cai et al. used AgCl as a template to prepare a hollow porous structure by the heteroepitaxial growth of Au nanocrystals on AgCl crystals and used them for the capture of hexachlorocyclohexane pesticides and ultra-sensitive detection based on SERS [39]. So far, various physical and chemical methods have been developed to prepare porous metal nanomaterials, including the template method, galvanic replacement, and dealloying method. However, although the template method can produce a controllable porous network with regular nanopore distribution, the sample prepared by the template method has a relatively low surface area. In addition, the template method usually requires the use of a large amount of organic ligand additives, organic polymers and reducing agents, thus the surface of the prepared nanomaterial adsorbs organic substances, thereby affecting its application. When the porous metal nanomaterial is prepared by the galvanic replacement reaction, due to the reducing atoms which often occupy only the outermost area randomly, the uniform porous structure cannot be prepared. Furthermore, dealloying methods have been widely used to synthesize porous metal nanomaterials. This method requires first synthesizing alloyed nanoparticles, and then selectively etching less-stable metals from the fully alloyed nanoparticles to produce porous noble metal nanostructures. In this process, calcination is usually required for synthesis, which requires high temperatures to obtain a clean surface. This makes the preparation process more complicated. Therefore, there is an urgent need to develop a simple and inexpensive method to prepare porous noble metal particles with controllable microstructures and clean and accessible surfaces.

**Figure 1:**
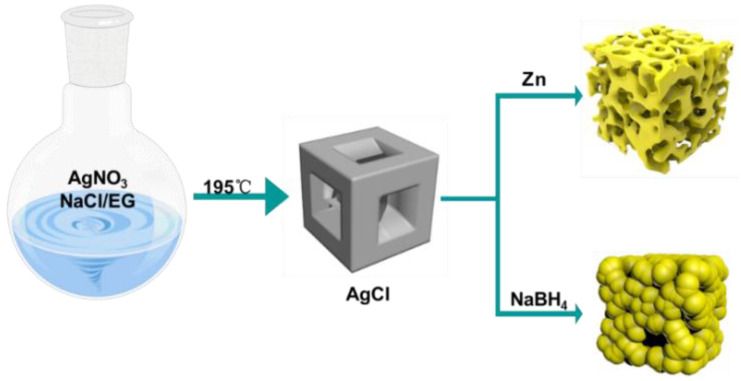
Schematic diagram of the preparation process for granular and sponge-like Ag cubes.

In this article, we report a simple, fast and inexpensive method to prepare granular porous Ag and sponge-like porous Ag with a highly accessible surface. As shown in Figure 1, the preparation process includes the preparation and reduction of AgCl. We first prepared AgCl cubes according to our previous reported method [40]. Additionally, we successfully prepared porous Ag cubes with a sponge-like structure through a galvanic replacement approach using sacrificial Zn and granular porous Ag cubes by optimizing the amount of reducing agent. Two kinds of porous Ag with different structures were prepared by reducing AgCl at room temperature without any surfactant, so they have a clean and highly accessible surface to target analysts. Furthermore, we used the porous Ag as a SERS substrate material to detect five kinds of methamphetamine analogs, including Ephedrine, Amphetamine, N-Methyl-1-(benzofuran-5-yl)propan-2-amine (5-MAPB), N-Methyl-1-(4-methoxyphenyl)propan-2-amine (PMMA) and N-Methyl-1-(4-fluorophenyl)propan-2-amine (4-FMA). Additionally, we realized the trace detection of the above five substances. Additionally, the Raman spectra of the above five substances were calculated by DFT, and the vibration peaks were assigned through the calculation results.

## 2. Results and Discussion

### 2.1. Characterization of the Prepared Porous Ag

**Figure 2:**
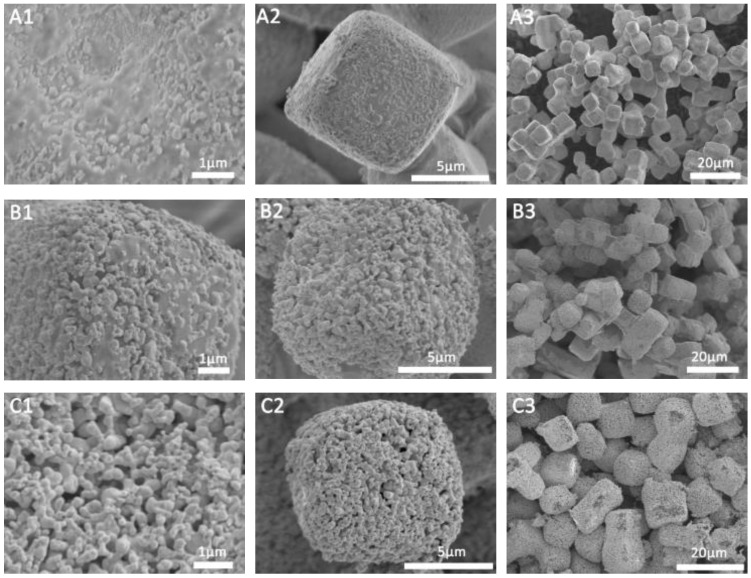
SEM images of different magnifications of porous Ag structure prepared when the ratio of NaBH4 to AgCl is 1:2 (**A1**–**A3**), 1:1 (**B1**–**B3**), 2:1 (**C1**–**C3**).

**Figure 3:**
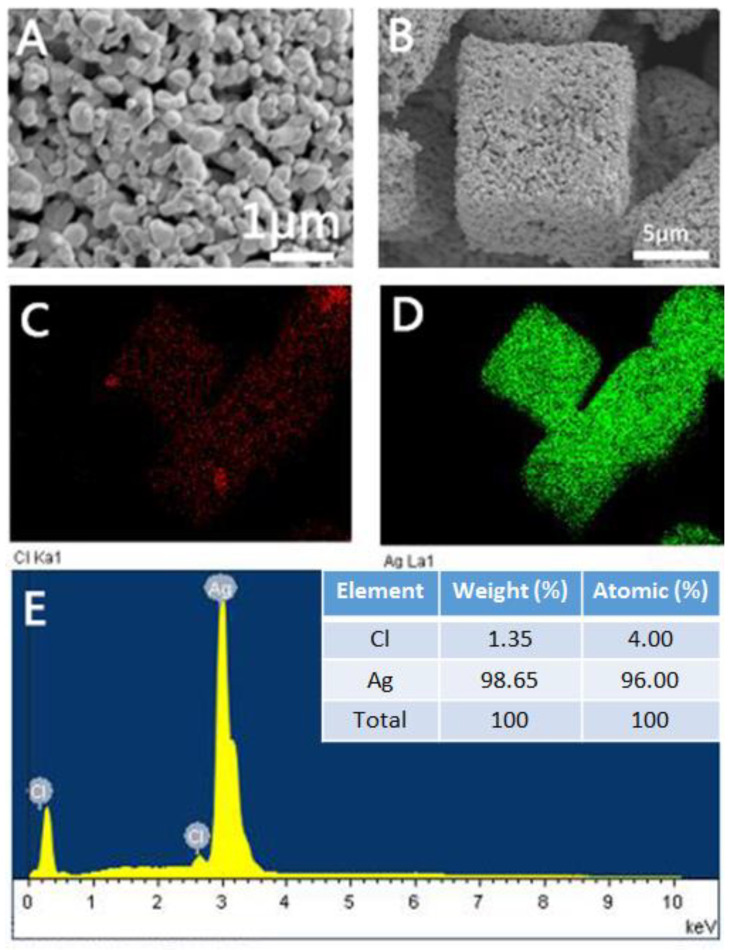
(**A,B**) SEM images of porous Ag structure prepared when the ratio of NaBH_4_ to AgCl is 2:1; (**C**,**D**) The distribution of Cl and Ag on the porous Ag surface, respectively; (**E**) Energy dispersive X-ray spectroscopy diagram of porous Ag particles.

In this article, we use AgCl as a sacrificial template to prepare a porous Ag microstructure. We first prepared concave Ag microcubes according to our previous reported method with a little modification [40]. Figure S1 shows the Scanning Electron Microscope (SEM) image of the AgCl microcubes. It can be clearly seen that the prepared microcubes have regular concave surfaces and sharp corners and edges. Additionally, the average size of the edge of the as prepared microcubes is approximately 10 μm. The as-prepared AgCl microcubes were used as templates to synthesize porous Ag particles by two different methods. First, we use NaBH_4_ as a reducing agent to reduce concave AgCl cubes to prepare porous Ag. We observed that when the reducing agent NaBH_4_ was added, the AgCl immediately changed from white to gray and was suspended in the solution. Figure 2 shows the SEM images of different magnifications of the products prepared by adding different amounts of NaBH_4_. As shown in Figure 2A1–A3, when the ratio of NaBH_4_ to AgCl is 1:2, we will prepare AgCl@Ag, and only a small amount of Ag is reduced on the AgCl surface, the main part is still AgCl. Moreover, when the ratio of NaBH_4_ to AgCl is 1:2, we further characterized the prepared AgCl@Ag. Figure S2C,D show the distribution of Cl and Ag on AgCl@Ag, respectively. From the EDS diagram of the prepared AgCl@Ag particles (Figure S2E), it can be seen that there is a large amount of AgCl. The atomic content ratio of Ag to Cl is 64: 36. When the amount of NaBH_4_ is increased so that the ratio of NaBH_4_ to AgCl is 1:1, the amount of Ag in the prepared AgCl@Ag will increase and the size of Ag particles anchored on the AgCl surface also increases, as shown in Figure 2B1–B3. When the content of NaBH_4_ increases to the ratio of NaBH_4_ to AgCl of 2:1, the granular porous Ag was formed, as shown in Figure 2C1–C3. To further characterize the prepared porous Ag structure, we can see the morphology of which remains cubic (Figure 3B). The high magnification SEM image (Figure 3A) shows that the produced Ag NPs are assembled into a 3D structure with a high rough surface and the Ag particles are filled with pore structures. From the EDS element distribution spectrum (Figure 3C,D) and element content (Figure 3E), it can be seen that there is almost no Cl element; that is, AgCl is all reduced to Ag, and finally the granular porous Ag cube is formed. Interestingly, we can see that the porous Ag particles formed after the reduction of concave AgCl cube, resulting in a regular cubic morphological structure with a flat surface. Therefore, we believe that during the reduction process, there will be a rearrangement process of Ag atoms, which causes the original concave surface to disappear and form a flat surface with lower energy.

In addition, we used Zn as a sacrificial template to prepare a porous Ag structure by reducing AgCl through a galvanic replacement reaction according to previous reports [41]. Figure S3A,B are the SEM images of the porous Ag structure formed after AgCl is completely reduced by Zn. We can see that the porous Ag maintains the cubic structure. Figure S3D,E show the distribution of Ag and Cl element of the porous Ag particles, respectively, and Figure S3F is the EDS diagram of porous Ag particles. It can be clearly seen that compared with sponge-like porous Ag particles, granular porous Ag has a rougher surface and deeper gaps, which make it an ideal highly reinforced SERS substrate material.

### 2.2. SERS Properties of the Sponge-like and Granular Porous Ag Cubes

**Figure 4:**
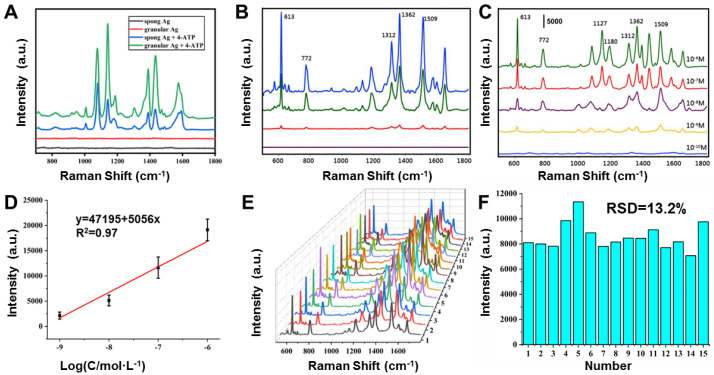
(**A**) Sponge and granular porous Ag used as a SERS substrate to detect 10^−6^ M 4-ATP. (**B**) Particles prepared at different ratios of NaBH_4_ to AgCl (purple curve 0, red curve 1:2, green curve 1:1, blue curve 2:1) were used to detect 10^−6^ M R6G. (**C**) Granular porous Ag used as a SERS substrate to detect R6G at 10^−6^ M–10^−10^ M. (**D**) Intensity of 613 cm^−1^ as a function of the concentrations of R6G. (**E**) SERS spectra of 15 different points of R6G. (**F**) Graphs of the intensity of the peaks at 613 cm^−1^ from 15 SERS spectra.

In order to prove that the prepared porous Ag base material is free of impurities, we first tested the prepared porous Ag base material. As shown in Figure 4A (red line and black line), when nothing is added, the porous Ag has no Raman scattering peak, indicating that the porous Ag structure itself is clean and will not interfere with the test. Then, we compared the SERS enhanced effect of spongy and granular porous Ag. As shown in Figure 4, we first added two kinds of Ag particles to 1.0 mL of 4-ATP solution with a concentration of 10^−6^ M and put it at room temperature for 1 h to allow 4-ATP molecules to adsorb on the Ag particles through Ag-S bound. Then, Ag particles were collected and dropped on a glass plate for the SERS test. It can be seen from Figure 4A that the SERS enhanced effect of granular porous Ag on 4-ATP was higher than that of spongy Ag, indicating that the granular porous Ag structure had a better SERS enhanced effect. In addition, we also investigated the SERS enhanced effect of AgCl under different reduction degrees. As shown in Figure 4B, AgCl with different reduction degrees was applied to detect R6G at 10^−6^ M. When there was no reducing agent, R6G could not be detected by AgCl as SERS substrate material. As AgCl was gradually reduced to Ag, the characteristic peak of R6G would be detected gradually. As the increase in Ag content, the characteristic peaks of R6G at 613, 772, 1312, 1362 and 1509 cm^−1^ gradually enhanced, indicating that its SERS enhanced effect was better with the increase in AgCl reduction degree. When AgCl was completely reduced to porous Ag, the SERS enhanced effect was the best. Therefore, we all adopted fully reduced porous Ag as SERS substrate material for detection in the subsequent tests. Figure 4C is the SERS spectrum of R6G with different concentrations. The Raman peaks at 613, 772, and 1180 cm^−1^ correspond to C-C-C in-plane vibration, C-H out-of-plane bending vibration, and C-H in-plane bending vibration in R6G, respectively. The Raman peaks at 1312, 1362 and 1509 cm^−1^ correspond to the N-H plane bending vibration and the C-C plane stretching in R6G, respectively [42]. Moreover, the characteristic peak strength of R6G increased significantly with the increase in the concentration, and the minimum detection concentration of R6G reached 10^−10^ M. According to previous reports [43,44], the EF value @613 cm^−1^ was calculated to be 4.3 × 10^6^. The detailed calculation process can be found in Supplementary Information S1. In addition, Figure 4D presents a linear calibration graph between the intensity of the Raman peak and the concentration of R6G. The y value represents the intensity of the Raman peak at 613 cm^−1^ and the x value represents the logarithm of the concentration of R6G. A linear regression equation is proposed. The reproducibility of the SERS signal is another important feature of the actual analysis. To test the reproducibility, we selected 15 random locations to collect R6G SERS signals. All spectra of the 15 random spots are shown in Figure 4E. The relative standard deviation (RSD) of the absolute intensity of the prominent peak at 613 cm^−1^ is calculated as 13.2%, indicating the good reproducibility of the SERS substrate.

**Figure 5:**
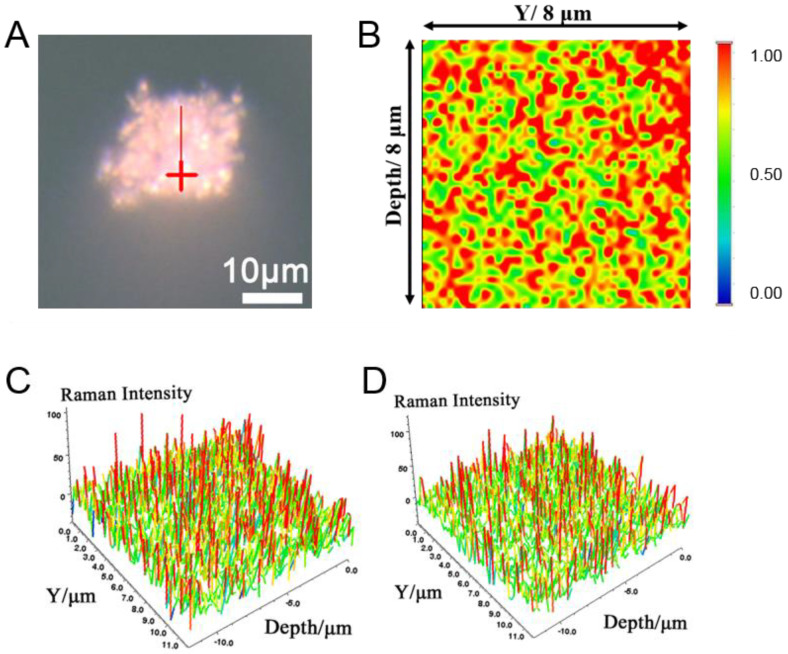
(**A**) Optical micrograph of a single particle of granular porous Ag. (**B**) Longitudinal Raman mapping along the red line in (**A**). (**C**) Distribution of the SERS intensity at the 613 cm^−1^ peak. (**D**) Distribution of the SERS intensity at the 1127 cm^−1^ peak.

We further explored the SERS hotspot distribution of granular porous Ag by Raman imaging. The porous Ag was soaked in 10^−4^ M R6G solution for 30 min, so that the R6G molecules were adsorbed on the surface of the porous Ag to form a monolayer, and then longitudinal scanning imaging was used to explore the SERS hot spot distribution inside the granular porous Ag, as shown in Figure 5. Figure 5A is an optical micrograph of a single granular porous silver and Figure 5B is the longitudinal SERS mapping along the red line in Figure 5A. From the figure, we can see that there are dense SERS hot spots distributed in the granular porous Ag. Figure 5C,D are the distribution diagrams of the SERS intensity at the peak positions of 613 cm^−1^ and 1127 cm^−1^, respectively. From the figure, we can also see that there are a large number of SERS-enhanced hot spots (the red curve part) on the longitudinal section inside the porous Ag. This indicates that a large number of dense SERS-enhanced hot spots distributed inside the granular porous Ag can be used for highly sensitive detection.

**Figure 6:**
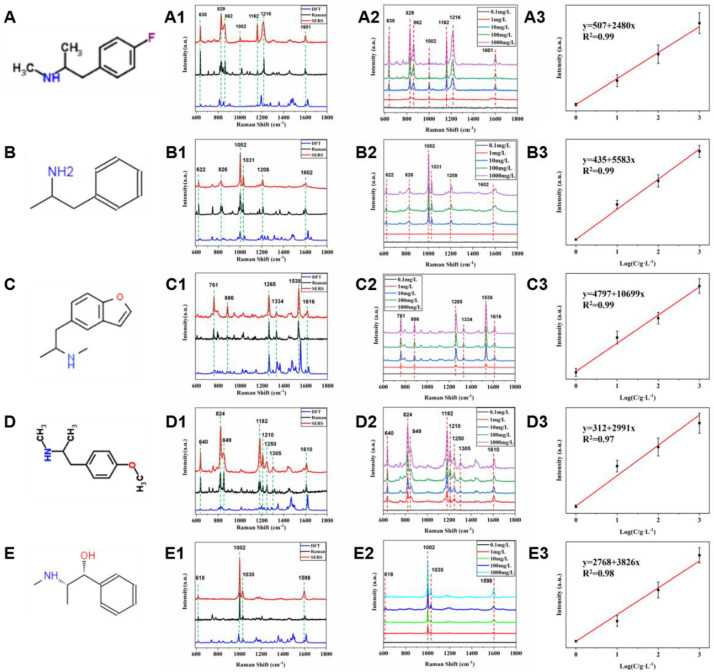
(**A**–**E**) Molecular structures of 5 kinds of amphetamine analogs, (**A1**–**E1**) Raman spectrum (black), DFT (blue) and SERS spectrum (red) of 5 kinds of amphetamine analogs powders, (**A2**–**E2**) SERS spectra of 5 kinds of amphetamine analogs at different concentrations. (**A3**–**E3**) The calibration curves of 5 kinds of amphetamine analogs.

As is well known, Illegal drug abuse is a common problem faced by all countries in the world. In particular, rapid and highly sensitive detection of drug abuse plays a crucial role in anti-drug studies. Surface-enhanced Raman spectroscopy (SERS) as a fast, non-destructive and highly sensitive detection technology has a good application prospect in the field of drug detection. In this article, we applied the porous Ag structure to the detection of five kinds of methamphetamine analogs, including Ephedrine, Amphetamine, 5-MAPB, PMMA and 4-FMA. Figure 6A–E is the molecular structure schematic diagram of five methamphetamine analogs, and it can be seen that five molecules all have similar amphetamine parent structures. We first performed Raman tests on standards of five kinds of methamphetamine analogs, as shown by the black spectral curves in Figure 6A1–E1. Furthermore, we performed SERS tests on the hydrochloric acid solution of five methamphetamine analogs at 2 g/L, using granular porous silver as the base material, as shown in the red spectral curves in Figure 6A1–E1. From its Raman spectrum and SERS spectrum, we can see that the Raman peak positions of the two are basically consistent.

In addition, we calculated the Raman spectra of the above substances by DFT and assigned their characteristic peaks, thus realizing the identification of five methamphetamine analogs with similar structures. The structure of the above molecules was optimized, and the optimized molecules were calculated by DFT using B3LYP/6–311++G(d, p) [45,46,47]. In order to correct for errors caused by the harmonic approximation in the theoretical model, a scaling factor of was applied to the fundamental frequency [48]. From Figure 6A1–E1, it can be seen that the Raman characteristic peaks of 4-FMA are located at 638, 829, 862, 1002, 1162, 1216 and 1601 cm^−1^, and the Raman characteristic peaks of Amphetamine are located at 622, 826, 862, 1002, 1031, 1208 and 1602 cm^−1^; the Raman characteristic peaks of 5-MAPB are located at 761, 886, 862, 1265, 1334, 1538 and 1616 cm^−1^, and the Raman characteristic peaks of 5-MAPB are located at 761, 886, 862, 1265, 1334, 1538 and 1616 cm^−1^; the Raman characteristic peaks of PMMA are located at 640, 824, 849, 1182, 1210, 1250 and 1610 cm^−1^, and the Raman characteristic peaks of Ephedrine are located at 618, 1002, 1030 and 1598 cm^−1^. Figure 6A2–E2 is SERS spectra of five alkaloids at different concentrations. We can see that the minimum detection concentration of five methamphetamine analogs can reach 1 g/L. Figure 6A3–E3 are linear calibration curves of 4-FMA, Amphetamine, 5-MAPB, PMMA and Ephedrine (with peak intensities at 829, 1002, 1182, 1538, and 1002 cm^−1^ as the vertical axis, respectively). As you can see from the figure, the above substances have good linear correlations in the range of 1 g/L to 1 mg/L, and the R^2^ are all greater than 0.97.

**Figure 7:**
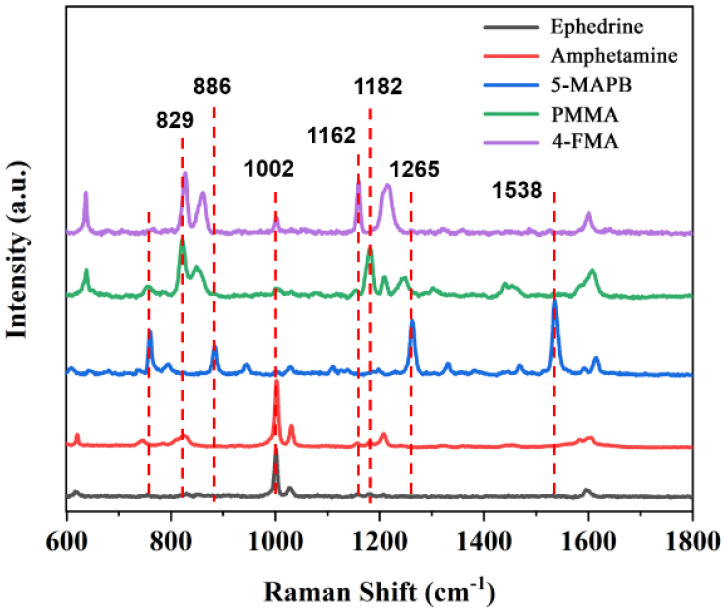
SERS spectra of Ephedrine, Amphetamine, 5-MAPB, PMMA and 4-FMA. The concentration is 2 g/L.

According to Figure 6A1–E1, we assigned the theoretical spectra, Raman spectra and SERS Raman peaks of the above five substances, as shown in Tables S1–S5. Raman spectroscopy is a molecular fingerprint spectroscopy that allows us to identify fentanyl and its analogues. Figure 7 shows the SERS spectra of five methamphetamine analogs. From Figure 7, we can see that when the analogs have only one group on the benzene ring (Ephedrine and Amphetamine), the breathing vibration of the benzene ring located at 1002 cm^−1^ and 1031 cm^−1^ has the highest intensity and is easier to be recognized. However, compared with ephedrine, amphetamine has an obvious Raman characteristic peak at 826 cm^−1^, which belongs to the stretching vibration mode of isopropyl C-C [49]. In addition, there are weak peaks around 1600 cm^−1^ for the five species, corresponding to the stretching and in-plane bending of the aromatic ring. In particular, 5-MAPB has a sharp and distinct Raman characteristic peak at 1538 cm^−1^ due to the presence of furan substituents. For PMMA and 4-FMA, which are very similar in structure, the only difference between the two is the substituents on the benzene ring. The biggest difference between the two Raman spectra is that the Raman vibration peak of C-F bond in 4-FMA is located at 1162 cm^−1^, while the Raman vibration peak of C-O bond in PMMA is located at 1182 cm^−1^.

## 3. Materials and Methods

### 3.1. Materials

All chemicals were purchased from commercial sources and used as received without further purification. Chemicals used in this study included silver nitrate (AgNO_3_, A.R. 99.8%, Sinopharm Group Reagent Co., Ltd., Shanghai, China), Hydrochloric acid (HCl, AR, 36.0–38.0%), Sodium chloride (NaCl, AR) nitric acid (HNO_3_, AR, 65.0–68.0%), ethylene glycol (EG, 99%), poly(diallyldimethylammonium) chloride (PDDA, MW = 200,000–350,000 D, 20 wt% in H_2_O, ≥99%), 4-aminothiophenol (4-ATP, 98% GC), Sodium borohydride (NaBH_4_, 98%) Rhodamine 6G (R6G, 95%), which were purchased from Aladdin (Shanghai, China). Zn tablets were purchased from Tengfeng Metal Company. 2-(methylamino)-1-phenylpropan-1-ol (Mw = 165.23), AmphetaMine (Mw = 135.2), 4-Fluoromethamphetamine (Mw = 167.22), 4-Methoxy Methamphetamine (Mw = 215.72) and 5-MAPB (Mw = 189.25) were purchased from The Third Research Institute of the Ministry of Public Security. The solutions were prepared from super pure water (18 MΩ cm) purified through a Milli-Q Lab system (Nihon Millipore Ltd., Shanghai, China).

### 3.2. Preparation of Concave AgCl Cube

The concave the AgCl cube was prepared according to our previous reported method with a little modification [40]. We took a 50 mL round bottom flask and add 20 mL of ethylene glycol, weighed 1.0 g of NaCl powder into the above ethylene glycol solution, stirred at room temperature to dissolve NaCl, then heated the above solution in an oil bath to 190 °C, and added 200 μL of 1 M AgNO_3_ solution. We continued the reaction for 30 min. A white precipitate appeared immediately after adding AgNO_3_ solution. After 30 min, the above solution was cooled to room temperature, centrifuged at 8000 rpm for 10 min, the supernatant was removed, 20 mL of water and ethanol were added to ultrasonically clean twice, and the obtained product was dispersed in ethanol for storage.

### 3.3. Preparation of Granular Porous Ag Cubes

The prepared AgCl was dispersed in 20 mL of pure water, added to a 100 mL beaker, and then uniformly dispersed by ultrasound at room temperature. Then, 0.2 M NaBH_4_ was added, reacted at room temperature for 30 min, and centrifuged at 8000 rpm for 10 min to remove the supernatant, and then washed twice with water and ethanol, and dispersed in ethanol for later use.

### 3.4. Preparation of Sponge-like Porous Ag Cube

We first cut the high-purity zinc foil into 5 × 5 cm^2^, cleaned the zinc foil with 0.1 M dilute nitric acid, and then rinsed with hydrated ethanol. Using the prepared AgCl cube as a template, we then added it to a zinc foil containing 50 mL of water with a pipette, and used a galvanic replacement reaction to reduce AgCl to obtain porous Ag. In addition, we adjusted the reaction time by adjusting the concentration of NaCl in the solution.

### 3.5. Instrumentation and Characterization

The prepared porous Ag crystals were characterized by scanning electron microscope (SEM, SU8010, Hitachi, Japan) and transmission electron microscope (TEM, Tecnai G2 F20 S-TWIN). The SEM study was conducted on a JEOL JSM-6700F SEM running at 3.0 kV. A Hitachi HT7700 operating at 100 kV was used for TEM characterization. Surface-enhanced Raman spectroscopy was performed using a confocal Raman microscope (Thermo Fisher DXR2xi) with a laser excitation at 633 nm, an exposure time of 10 s and an objective lens with a magnification of 50 times.

### 3.6. The SERS Test

A confocal Raman microscope (Thermo Fisher DXR2xi) was used for surface enhanced Raman detection under laser excitation at 633 nm, with a laser power of 6.0 mW and an acquisition time of 10 s. After concentrating the porous Ag separately, we took 10 μL and soaked them in 4-ATP solutions of different concentrations, and let them stand for 60 min at room temperature to allow 4-ATP molecules to adsorb on the surface of Ag crystals to form a monolayer, and then the crystals. The centrifugal collection was dripped on the glass slide for SERS detection. All the glass pieces used were soaked in aqua regia for 30 min, and then rinsed twice with ultrapure water and ethanol to remove impurities, and dried in an oven for later use. The 4-ATP concentration is 10^−6^ M. For the mapping of granular porous silver, 10^−4^ M R6G molecule was used as the test object, and the porous silver particles were soaked in the R6G solution for 30 min, and then the porous silver particles were dripped on the glass slide and dried at room temperature. The surface scan adopts 633 nm laser wavelength. The acquisition was performed three times and the acquisition time was set to 0.5 s. The step width of longitudinal Raman mapping is 0.2 μm, equipped with a microscope (50× objective lens). The SERS test conditions for five kinds of methamphetamine analogs are consistent with the above process, except that a 10× objective was used. For the detection of five kinds of methamphetamine analogs, including Ephedrine, Amphetamine, 5-MAPB, PMMA and 4-FMA, we first mixed 10 μL of the prepared porous Ag with 10 μL of the test solution and then performed the test after the solvent evaporated at room temperature.

## 4. Conclusions

We used AgCl cubes as the sacrificial template to prepare two kinds of porous Ag crystals. Using NaBH_4_ as the reducing agent, we prepared granular porous Ag cubes using the hydrothermal method. Using Zn as the sacrificial metal, we prepared sponge-like porous Ag cubes by a galvanic replacement approach. The two kinds of prepared porous Ag cubes were further used for SERS detection, and the results showed that the granular porous Ag cube has a better SERS effect than sponge porous Ag. Furthermore, we used granular porous Ag as the SERS base material to detect the five kinds of methamphetamine analogs, including Ephedrine, Amphetamine, 5-MAPB, PMMA and 4-FMA, with the lowest detection concentrations of 1 mg/L. Furthermore, we combined the analysis of the conventional Raman spectra, DFT calculated spectra and obtained SERS spectra of five kinds of methamphetamine analogs, and we achieved their discrimination. We showed that the porous Ag structure has great application prospects in label-free SERS detection.

## Supplementary Materials

The following supporting information can be downloaded at: https://www.mdpi.com/article/10.3390/molecules27123939/s1, S1: Calculation of EF value; Figure S1: SEM images of as prepared concave AgCl micro cubes. (A) High magnification, (B) Low magnification. Figure S2: (A) and (B) SEM images of porous Ag structure prepared when the ratio of NaBH4 to AgCl is 1:2; (C) and (D) are the distribution of Cl and Ag on the porous Ag surface, respectively; (E) EDS diagram of porous Ag particles. Figure S3: (A), (B) and (C) are SEM images of porous Ag structure prepared by galvanic replacement reaction, (D) and (E) are the distribution of Ag and Cl on the porous Ag surface, respectively. (F) EDS diagram of porous Ag particles. Tables S1–S5: Expiremental and DFT frequencies with respective spectral assignments for 5 kinds of methamphetamine analogs.

## Sample Availability

Samples of the crystals are available from the authors. Samples of the drugs are not available from the authors.
