# Supplementary material for: Facile Synthesis of Porous Ag Crystals as SERS Sensor for Detection of Five Methamphetamine Analogs
Source: Molecules. 2022 Jun 20;27(12):3939. doi: 10.3390/molecules27123939 (PMC9227489; doi:10.3390/molecules27123939)
Supplement: Supplementary file 1 [file molecules-27-03939-s001.zip › molecules-1760347-supplementary.pdf]

Electronic Supplementary Material (ESI)

Facile synthesis of porous Ag crystals as SERS sensor for detection of 5 methamphetamine analogs

Yazhou Qin[a], Mingjie Chen[a], Shusheng Yin[a], Yuanzhao Wu [a], Yingsheng He*[b] and Weixuan Yao*[a]

a Key Laboratory of Drug Prevention and Control Technology of Zhejiang Province Zhejiang Police College, 555 Binwen Road, Binjiang District, Hangzhou 310053, Zhejiang Province, P. R. China.

b Key Laboratory of Drug Control and Monitoring, National Anti-Drug Laboratory Zhejiang Regional Center. 555 Binwen Road, Binjiang District, Hangzhou 310053, Zhejiang Province, P. R. China.

* Corresponding authors.

E-mail addresses: 21537047@zju.edu.cn (Y. S. He), yaoweixuan@zjjcxy.cn (W. X. Yao)

S1. Calculation of EF value

The *EF* value can be calculated by the following equation:

$$EF = (I_{SERS} / I_{bulk}) (N_{bulk} / N_{SERS}) \quad (1)$$

where I_{SERS} is the intensity of the Raman spectra of the sample. I_{bulk} is the Intensity of the normal Raman spectra of solid R6G. N_{bulk} is the molecule number of the solid R6G in the laser illumination volume. N_{SERS} is the total number of surface adsorbed molecules. Taking the 613 cm^{-1} peak as an example, Ibulk and ISERS were measured to be 75 and 23478 counts (respectively). $\rho_{R6G} = 0.99$ g/cm^3 N_{bulk} was about 2×10^9. The laser penetration depth is about 2 microns and the diameter is about 1 micron. The collection area of the R6G is about 12.5 mm^2. The volume of the R6G solution is 10 microliters. Thus, N_{SERS} is about 3.78×10^5.

$N_{SERS} = N_A \times 0.25\pi\ \mu m^2 \times (10\ \mu L \times 10^{-6}\ M)/(12.5\ mm^2) = 3.78 \times 10^5$

Thus, the *EF* value @613 cm^{-1} was calculated to be 4.3×10^6.

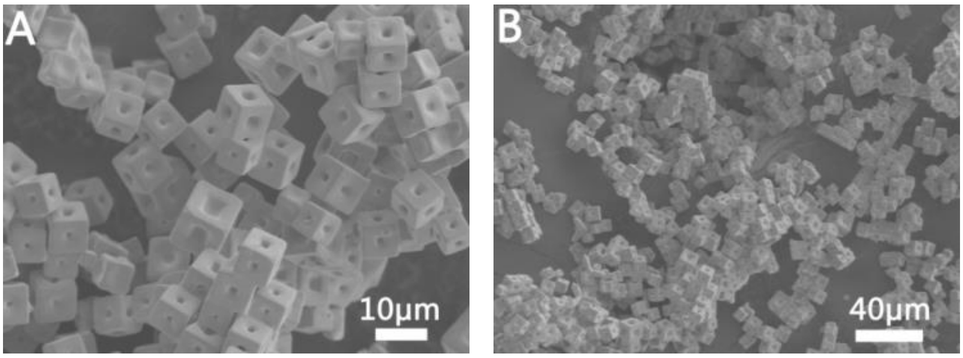

Figure S1. SEM images of as prepared concave AgCl micro cubes. (A) High magnification, (B) Low magnification.

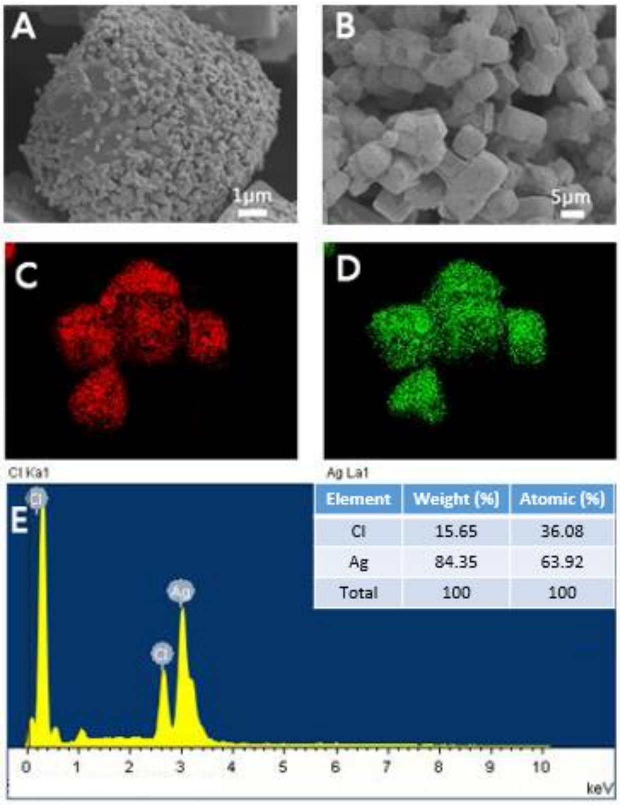

Figure S2. (A) and (B) SEM images of porous Ag structure prepared when the ratio of $NaBH_4$ to AgCl is 1:2; (C) and (D) are the distribution of Cl and Ag on the porous Ag surface respectively; (E) EDS diagram of porous Ag particles.

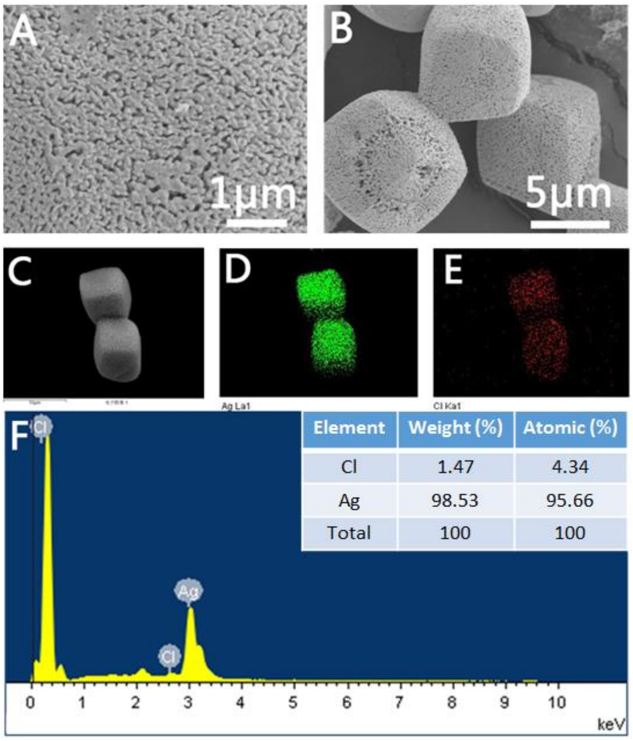

Figure S3. (A), (B) and (C) are SEM images of porous Ag structure prepared by galvanic replacement reaction, (D) and (E) are the distribution of Ag and Cl on the porous Ag surface respectively. (F) EDS diagram of porous Ag particles.

Table S1. Expiremental and DFT frequencies with respective spectral assignments for Ephedrine.

Ephedrine	Raman/cm^{-1}	SERS/cm^{-1}	DFT x 0.9894/cm^{-1}
δ(ring),δ(C-C)	621	618	620
β(ring)	1003	1002	993
ν (C=C)	1034	1030	1031
ν (C=C),$\varrho(C\text{-}H)_{benzene}$	1606	1598	1615

Table S2. Expiremental and DFT frequencies with respective spectral assignments for Amphetamine.

Amphetamine	Raman/cm^{-1}	SERS/cm^{-1}	DFT x 0.9970/cm^{-1}
δ(ring),δ(C-C)	624	622	632
δ(ring),ν(C-C),ν(C-N)	828	826	835
β(ring)	1003	1002	1001
ν(C-C),ν(C-N),ϱ(N-H)	1030	1031	1041
ν(C-C),ϱ(N-H),ϱ(C-H)	1212	1208	1228
ν (C = C),ϱ(C-H)$_{benzene}$	1601	1602	1624

Table S3. Expiremental and DFT frequencies with respective spectral assignments for 5-MAPB.

5-MAPB	Raman/cm^{-1}	SERS/cm^{-1}	DFT x 0.9937/cm^{-1}
γ(ring)$_{benzene}$,ϱ(N-H),γ(ring)$_{furane}$	759	761	764
ϱ(CH_2),ϱ(CH_3)	884	886	884
β(ring)$_{benzene}$,β(ring)$_{furane}$	1266	1265	1266
ν (C = C)$_{furane}$,ϱ(C-H)	1333	1334	1338
ν (C = C)$_{furane}$	1535	1538	1557
ν (C = C)$_{benzene}$,ϱ(C-H)$_{benzene}$	1616	1616	1626

Table S4. Expiremental and DFT frequencies with respective spectral assignments for 4-FMA.

4-FMA	Raman/cm^{-1}	SERS/cm^{-1}	DFT x 0.9944/cm^{-1}
δ(ring),δ(C-C)	638	638	641
ω(C-H)$_{benzene}$	829	829	814
δ(ring),ϱ(C-C),ϱ(C-O),ν(C-F)	865	862	852
ν(C-C)	1018	1005	1011
ϱ(C-H)$_{benzene}$	1161	1159	1163
β(ring)	1222	1216	1228
ν (C = C)$_{benzene}$,ϱ(C-H)$_{benzene}$	1600	1601	1600

Table S5. Expiremental and DFT frequencies with respective spectral assignments for PMMA.

PMMA	Raman/cm^{-1}	SERS/cm^{-1}	DFT x 0.9845/cm^{-1}
δ(ring)	639	640	636
γ(ring) $_{benzene}$, ν(C-C)	824	824	812
γ(ring) $_{benzene}$, ν(C-C)	853	849	841
ϱ(C-H) $_{benzene}$, ϱ(C-O)	1187	1182	1178
β(ring)	1211	1210	1217
ω(C-H)	1249	1250	1253
ν(C-O),β(ring) $_{benzene}$	1305	1305	1304
ν (C = C) $_{benzene}$,ϱ(C-H)$_{benzene}$	1609	1610	1622
